# Transcriptional upregulation of galectin-3 in multiple sclerosis

**DOI:** 10.1007/s12026-023-09408-x

**Published:** 2023-07-25

**Authors:** Katia Mangano, Maria Cristina Petralia, Rita Bella, Manuela Pennisi, José Francisco Muñoz-Valle, Jorge Hernández-Bello, Ferdinando Nicoletti, Paolo Fagone

**Affiliations:** 1 Department of Biomedical and Biotechnological Sciences, University of Catania, Via S. Sofia 89, 95123 Catania, Italy; 2 Department of Clinical and Experimental Medicine, University of Messina, 98122 Messina, Italy; 3 Department of Medical and Surgical Sciences and Advanced Technologies, University of Catania, Via S. Sofia 78, 95123 Catania, Italy; 4 Institute for Research in Biomedical Sciences, University Center for Health Sciences, University of Guadalajara, Guadalajara, Mexico

**Keywords:** Multiple sclerosis, Galectins, Galectin-3

## Abstract

Multiple sclerosis (MS) is an autoimmune, demyelinating disorder of the central nervous system (CNS) affecting approximately 2.5 million people worldwide. The mechanisms underlying the pathogenesis of MS are still only partially elucidated. Galectins are a family of β-galactoside-binding lectins that are involved in the regulation of immune and inflammatory responses and have been shown to exert a role in the maintenance of central nervous system (CNS) homeostasis. There has been an increasing interest in the role of galectin-3 in neuroinflammation and neurodegeneration. In the current study, we have evaluated the expression levels of galectin-3 in different cellular populations involved in the etiopathogenesis of MS. We have observed dramatically higher transcriptomic levels of galectin-3 in encephalitogenic CD4+ T cells in a preclinical model of MS, the MOG-induced experimental allergic encephalomyelitis (EAE). Also, significantly higher levels of galectin-3 were found in microglial cells, astrocytes, and oligodendrocytes isolated from the spinal cord of EAE mice, as well as in human MS-related white matter lesions. Modular co-expression analysis revealed that galectin-3 is co-expressed with genes involved in the regulation of microglia, cytokine production, and chemotaxis. This is the first comprehensive analysis of the expression of galectin-3 in MS, further strengthening its potential pathogenetic role in the etiopathogenesis of this CNS autoimmune disorder.

**Supplementary Information:**

The online version contains supplementary material available at 10.1007/s12026-023-09408-x.

## Introduction

Multiple sclerosis (MS) is an autoimmune, demyelinating disorder of the central nervous system (CNS), affecting approximately 2.5 million people worldwide [1, 2]. The most common clinical form is relapsing-remitting MS, representing about 85% of the cases. After a period ranging from 10 to 20 years, approximately 60% of the patients enter a phase that is characterized by a steady decline of neurological functions (secondary progressive MS, SPMS). Primary progressive MS (PP-MS) affects a subset of patients (10–15%) that is characterized by continuous progression of the disease from its onset. Current treatments (i.e., disease-modifying drugs) reduce the number and severity of relapses in RR-MS, but are ineffective in halting disease progression. Hence, there is a strong need to develop new therapeutic strategies for MS.

Galectins are a conserved family of β-galactoside-binding lectins, implicated in a variety of biological processes, e.g., adhesion and motility, cell survival and proliferation, angiogenesis, and inflammation [3]. Galectins contain either one or two carbohydrate recognition domains (CRDs) and can be found in both the cytosol and nucleus, as well as in the extracellular compartment, as they are secreted by a non-classical secretion process [3]. A growing body of pieces of evidence suggests that galectins, including galectin-1 and galectin-3, are involved in the regulation of CNS homeostasis and neuroinflammation by controlling microglial activation, inhibiting neurodegeneration, and promoting neuroprotection [4]. Galectin-1, galectin-3, galectin-4, galectin-8, and galectin-9 are expressed at high levels in the brain [5]; however, most studies on CNS function and protection have been dedicated to galectin-1 and galectin-3 [6].

There has been increasing interest in the role of galectin-3 (Lgals3) in neuroinflammation and neurodegeneration [7]. In particular, it was shown that the deletion of the galectin-3 gene improves experimental allergic encephalomyelitis (EAE) in mice [8] and was associated with a decreased Th17 and an increased regulatory T-cell response [8]. In another model, Wallerian degeneration following the injury of the sciatic nerve in galectin-3-deficient mice was associated with a significant increase in the inflammatory cytokines, IL-1β and TNF-α, and with the upregulation of toll-like receptors (TLR)-2 and -4 [9]. On the other hand, *Lgals3*^−/−^ mice, despite having a similar susceptibility to cuprizone-induced demyelination as wild-type mice, have an impaired efficiency of remyelination [10]. However, in another study, during cuprizone-induced demyelination in *Lgals3*^−/−^ mice, oligodendrocyte precursor cell (OPC) maturation was not affected by the deficiency of galectin-3 [11].

Interestingly, it has been reported the presence of auto-antibodies against galectin-3 in the sera from patients with secondary progressive multiple sclerosis (SPMS), which could be partly responsible for the progressive damage to the blood-brain barrier (BBB) associated with MS [12]. Moreover, the inhibition of the expression of galectin-3 in the brain microvascular endothelial human cells (BMEC) triggered an increase in the expression of intracellular adhesion molecule 1 (ICAM-1), responsible for the extravasation of leukocytes to the CNS [12]. Overall, the authors proposed that galectin-3 could drive a downregulation of ICAM-1 in BMEC, thus exerting a protective effect in MS [12].

In the present study, we aimed to characterize the transcriptional levels of galectin-3, along with the expression levels of the other galectin gene family members, in the different cell populations that are involved in MS pathology.

## Materials and methods

### Ex vivo study

#### Animals

The 8-to-10-week-old female C57BL/6 mice (Harlan Laboratories, San Pietro al Natisone, Udine, Italy) were housed in a controlled environment and provided with standard rodent chow and water. Animal care was in compliance with local regulations on the protection of animals used for experimental and other scientific purposes (Directive 86/609/EEC, enforced by the Italian D.L. No. 116 of January 27, 1992).

#### MOG-induced EAE in / 6 mice

The mice were immunized by subcutaneous injections of 200 μg myelin oligodendrocyte glycoprotein (MOG) (35–55) (Genemed Synthesis, San Francisco, CA) in complete Freund’s adjuvant, containing 1 mg M. tuberculosis, strain H37RA (CFA; Difco, Detroit, MI), divided among two sites draining into the axillary lymph nodes. Sham mice, that served as negative controls, were injected with complete Freund’s adjuvant without MOG (35-55). After immunization (day 0) and on day 2, the mice received intraperitoneal injections of 200 ng pertussis toxin (List Biological Laboratories, Campbell, CA) dissolved in phosphate-buffered saline (PBS).

#### Cell preparation

The animals were observed daily to evaluate the clinical signs of EAE, and when the peak of the disease was reached (mean score ± S.D. 2.5 ± 0.038), the spleen and spinal cords were harvested from the mice. Cell suspensions from the spleen were prepared by grinding the organs with the plunger of a 5-ml disposable syringe and suspending them in RPMI 1640 medium (Sigma, Milan, Italy) supplemented with 10% fetal calf serum, 2 mM glutamine, and 50 mg/ml of penicillin/streptomycin. Splenocytes were then treated with an ACK lysis buffer (Invitrogen, Life Technologies Italia, Monza, Italy) to remove red blood cells. Single-cell suspensions from spinal cords were obtained using commercially available kits (Miltenyi) by mechanical dissociation and enzymatic degradation, followed by magnetic cell sorting using anti-ACSA-2 (for astrocytes), anti-O4 (for oligodendrocytes), anti-CD11b (for microglia), and anti-CD4 (for infiltrating T cells) antibodies (Miltenyi).

#### RNA isolation

Total RNA was isolated using the RNeasy® Mini Kit (Quiagen) according to the manufacturer’s protocol. Isolated RNA was further cleaned of possible genomic DNA by treatment with DNase I (Roche). The quality and integrity of total RNA preparation was confirmed using a NanoDrop™ 2000c Spectrophotometer (Thermo Scientific). cDNA synthesis was performed by reverse transcription of total RNA using the SuperScript® III First Strand Synthesis System and random hexamer primers (Invitrogen), following the manufacturer’s instructions.

#### Quantification of gene expression by real-time PCR

Primer sequences were designed in-house or downloaded from PrimerBank (https://pga.mgh.harvard.edu/primerbank/). Sequences for primers are listed in Table 1. Real-time PCR was performed using the QuantiNova SYBR Green mastermix (Qiagen). Gene expression was calculated using the formula: 2−^ΔΔCt^, where ΔΔCt = (Ct_target gene_ − Ct_GAPDH_) _EAE_ – (Ct_target gene_ − Ct_GAPDH_)_control_.

**Table 1:** Primers used in this study

GAPDH forward	TGGATTTGGACGCATTGGTC
GAPDH reverse	TTTGCACTGGTACGTGTTGAT
Lgals3 forward	AGACAGCTTTTCGCTTAACGA
Lgals3 reverse	GGGTAGGCACTAGGAGGAGC
Lgals3bp forward	TGCTGGTTCCAGGGACTCAA
Lgals3bp reverse	CCACCGGCCTCTGTAGAAGA
Lgals1 forward	AACCTGGGGAATGTCTCAAAGT
Lgals1 reverse	GGTGATGCACACCTCTGTGA
Lgals12 forward	CCTCCTGGGGACGAAAGAAG
Lgals12 reverse	GGACACAGTAGAGGTGGACAT
Lgals2 forward	AACATGAAACCAGGGATGTCC
Lgals2 reverse	CGAGGGTTAAAATGCAGGTTGAG
Lgals4 forward	TGGGTACAACCCTCCACAGAT
Lgals4 reverse	ATATGGCACACGCGGATTGAA
Lgals6 forward	TCTTCAACACGAAGCAAAGCG
Lgals6 reverse	CTGTCATGGCCGGATATTTGG
Lgals7 forward	ATGTCTGCTACCCAGCACAAG
Lgals7 reverse	GTCCAGCCTCGGGTTAAAGT
Lgals8 forward	ATAATCCCCTATGTTGGCACCA
Lgals8 reverse	TGAACCGAGGGTTAAAGTGGAA
Lgals9 forward	ATGCCCTTTGAGCTTTGCTTC
Lgals9 reverse	AACTGGACTGGCTGAGAGAAC
Lgals12 forward	CCTCCTGGGGACGAAAGAAG
Lgals12 reverse	GGACACAGTAGAGGTGGACAT

### In silico data

The NCBI Gene Expression Omnibus (GEO) database (http://www.ncbi.nlm.nih.gov/geo/ (accessed on 20 July 2022)) was used to identify transcriptomic datasets that included samples from MS patients.

#### Analysis of Lgals3 expression in MS lesions

The GSE108000 microarray dataset included 7 chronic active MS lesions, 8 inactive MS lesions, and white matter of 10 control donors [13]. The dataset was generated using the Agilent-026652 Whole Human Genome Microarray 4x44K v2 platform. Raw data were quantified using Agilent Feature Extraction software and normalized using loess, followed by a between-array normalization using the Gquantile algorithm in LIMMA. Details on the experimental design of the dataset can be retrieved from the associated publication [13].

### Statistical analysis

The ex vivo results are shown as means ± standard error of the mean (S.E.M.). Data were subjected to the Shapiro-Wilk and Kolmogorov normality test, and based on the results, either parametric tests (Student’s T test or ANOVA) or non-parametric tests (Mann-Whitney or Kruskal-Wallis test) were used to assess the differences between the EAE and Sham mice. Values of *p* < 0.05 were considered statistically significant. The statistical analysis of the microarray data was performed using the LIMMA (linear model for microarray analysis) test. An adjusted (Benjamini–Hochberg corrected) *p*-value (adj. *p*-value, FDR: false discovery rate) <0.05 was considered as a threshold for statistical significance. The submitter-supplied gene expression matrix was used for the analysis. The multiple-probe collapse was performed by the sum method. Hierarchical clustering and gene similarity matrix were calculated using the Spearman correlation as similarity metrics. Modular gene co-expression network analysis was performed using the CEMiTool software [14], using default settings. Gene ontology and pathway analysis was performed using the online software, Metascape [15].

## Results

### Lgals3 is overexpressed in the spinal cord of EAE mice

**Fig. 1:**
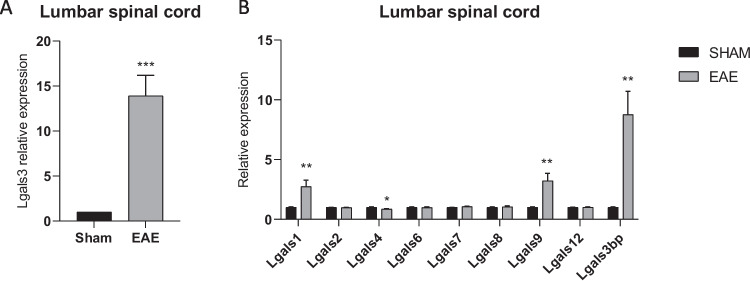
*Galectin-3* expression in the spinal cord from a model of multiple sclerosis. (**A**) Transcriptomic levels of *galectin-3* in the spinal cord in mice affected from MOG-induced EAE. (**B**) Expression of the galectin gene family members in the spinal cord in mice affected from MOG-induced EAE; *p < 0.05; **p < 0.01; ***p < 0.001

We have investigated the expression levels of galectin-3 in the lumbar spinal cord from an animal model of MS, the MOG-induced EAE. As shown in Fig. 1A, significantly higher levels of *Lgals3* are found in EAE spinal cord, as compared to the spinal cord collected from control (sham) mice (Fig. 1A). Moreover, a significant upregulation was observed for *Lgals1*, *Lgals9*, and *Lgals3bp* (Fig. 1B), while a moderate but significant downregulation was observed for *Lgals4* (Fig. 1B).

### Evaluation of galectin-3 expression in CNS populations

**Fig. 2:**
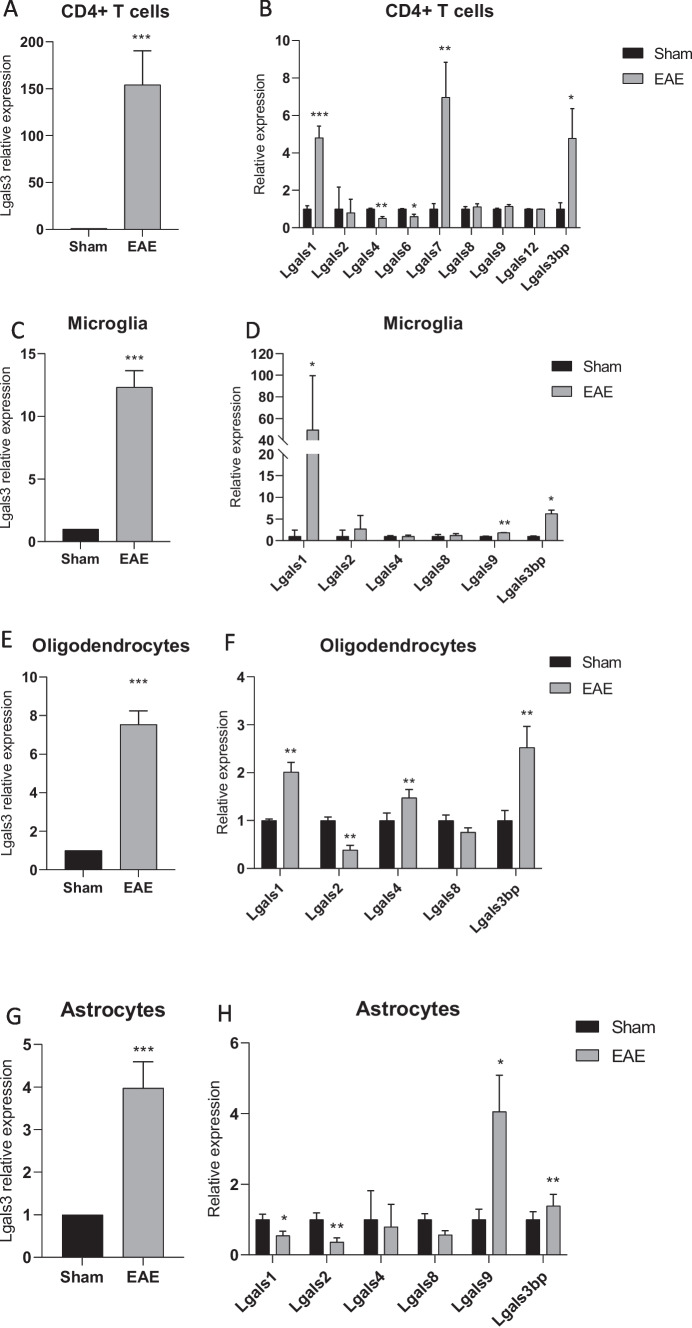
*Galectin-3* expression in the CNS cell populations from a preclinical model of multiple sclerosis. (**A**) Expression levels of *galectin-3* in encephalitogenic CD4+T cells isolated from the spinal cord of mice with MOG-induced EAE and CD4+ cells from the spleen of healthy control mice. (**B**) Expression levels of galectin gene family members in encephalitogenic CD4+ T cells isolated from the spinal cord of mice with MOG-induced EAE and CD4+ cells from the spleen of healthy control mice. (**C**) Expression of *galectin-3* in the microglia of mice with MOG-induced EAE. (**D**) Expression levels of galectins in the microglia of mice with MOG-induced EAE. (**E**) Expression of *galectin-3* in oligodendrocytes isolated from MOG-induced EAE mice. (**F**) Expression levels of galectins in oligodendrocytes isolated from MOG-induced EAE mice. (**E**) Modulation of *galectin-3* in astrocytes isolated from MOG-induced EAE mice. (**F**) Expression of the galectin gene family members in astrocytes isolated from MOG-induced EAE mice; *p < 0.05; **p < 0.01; ***p < 0.001

To better characterize the contribution of each CNS cellular population to the increased expression of *galectin-3*, we investigated the expression levels of *Lgals3*, and of the other members of the galectin gene family, in sorted CNS populations from EAE mice and unimmunized control animals. As shown in Fig. 2A, significantly higher levels of *Lgals3* are found in CNS-infiltrating T helper cells, as compared to spleen CD4+ T cells isolated from sham mice (Fig. 2A). As shown in Fig. 2B, a strong upregulation was also observed for *Lgals1*, *Lgals7*, and *Lgals3bp* (Fig. 2B). High-throughput data from an independent study [16] confirmed our observations of an increase in *Lgals3* expression in CNS-infiltrating CD4+ T cell in a MOG-induced EAE model and, in addition, provided evidence for a not significant trend of upregulation in *Lgals3* levels in spleen CD4+T cells isolated from EAE mice, as compared to those isolated from naïve mice (Supplementary Fig. S1).

Along the same lines, we observed a significant upregulation of *Lgals3* in the microglia (Fig. 2C) and in the oligodendrocytes isolated from EAE mice, as compared to healthy mice (Fig. 2E). A significant increase in *Lgals1* and *Lgals3bp* was observed in both microglial cells (Fig. 2D) and oligodendrocytes (Fig. 2F). Similarly, a significant increase in *galectin-3* transcriptomic levels was found in the astrocytes isolated from the spinal cord of EAE mice (Fig. 2G). Finally, a marked increase was also observed in astrocytes for *Lgals9* and *Lgals3b*, along with a significant downregulation of *Lgals1* and *Lgals2* (Fig. 2G).

### Galectin-3 in MS lesions

**Fig. 3:**
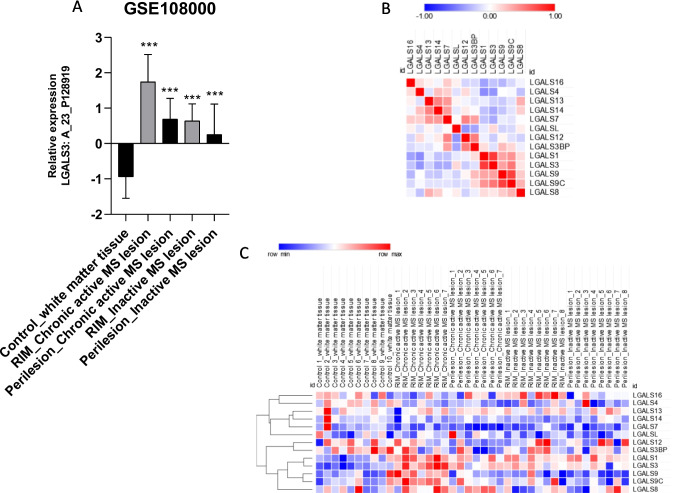
Galectin-3 in multiple sclerosis patients’ lesions. (**A**) Expression of galectin-3 white matter lesions from multiple sclerosis patients, as determined from the GSE108000 dataset. (**B**) Similarity matrix for galectin expression levels from the GSE108000 dataset. (**C**) Heatmap of galectin gene expression levels, determined in the GSE108000 dataset. The expression level of each gene is color-coded from blue (lower expression) to red (higher expression); *FDR < 0.05; **FDR < 0.01; ***FDR < 0.001

By interrogating the publicly available GSE108000 dataset, we constructed the expression profile of the galectin gene family members in normal white matter, chronic active MS lesions (representing the early events in demyelination), in the rim of chronic active MS lesions (i.e., fully active demyelination), in the rim of inactive MS lesions (halt of demyelination), and in inactive lesions (absence or suppression of early demyelination). As compared to the normal white matter, MS lesions were characterized from a significant upregulation of *Lgals3* expression (Fig. 3A). A similar trend of modulation was observed for *Lgals3bp*, *Lgals1*, *Lgals9*, and *Lgals8* (Fig. 3B,C).

**Fig. 4:**
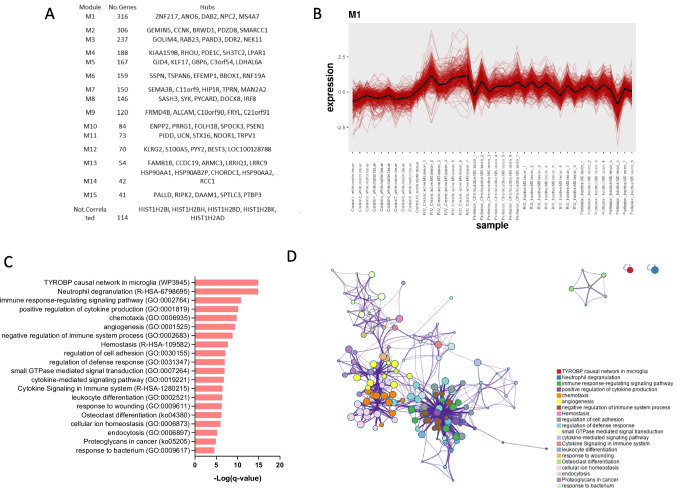
Biological interpretation of *Lgasl3* expression in multiple sclerosis lesions. (**A**) Results from the modular gene co-expression network analysis performed on the data from the GSE108000 dataset. (**B**) Co-expressed genes belonging to the M1 cluster. (**C**) Gene ontology and pathway analysis of the genes belonging to the M1 cluster. (**D**) Network showing the connection among the pathways enriched by the genes belonging to the M1 cluster

To gain further insight into the biological role of galectin-3 in MS lesions, we performed a modular co-expression analysis, which identified *Lgals3* to belong to the M1 cluster (Fig. 4A,B). The M1 cluster included 316 genes, with *ZNF217*, *ANO6*, *DAB2*, *NPC2*, and *MS4A7* being the network hub genes (Fig. 4A). Gene ontology and pathway analysis revealed that the genes belonging to the M1 cluster are involved in several biological processes, such as those related to microglia, neutrophil degranulation, cytokine production, and chemotaxis (Fig. 4C,D).

## Discussion

Galectin-3 is the unique representative of the chimera-type galectins, containing one CRD linked to the N-terminal domain, which allows its oligomerization and the formation of pentamers [17]. Galectin-3 has a molecular weight of 31 kDa, and it was first identified in immune cells and subsequently found in a series of normal and cancer cells. The structure of galectin-3 consists of two structurally specific domains: the N-terminal domain and the C-terminal CRD. The N-terminal domain contains sites for phosphorylation involved in the regulation of secretion, while the C-terminal CRD is connected to a collagen-like sequence, assembled of proline, glycine, and tyrosine tandem repeats [17]. There are several ligands for galectin-3, both in the intracellular and the extracellular space, such as Bcl-2, synexin, and β-catenin, inside the cells, and laminin, fibronectin, CD29, CD66, α1β1 integrin, and galectin-3-binding protein (Lgals3bp) in the extracellular space [17].

In the present study, we have evaluated the expression levels of *galectin-3* in cell populations that are involved in the pathogenesis of MS. We have also provided data on the modulation of the other members of the galectin gene family for comparison.

Our data show a significant increase in *galectin-3* expression in encephalitogenic CD4+ T cells in a preclinical model of MS. Also, significantly higher levels of *galectin-3* were found in microglial cells, astrocytes, and oligodendrocytes, both in MOG-induced EAE and in human MS-related white matter lesions.

Galectin-3 has been shown to be expressed in neuronal and glial cells [18]. The nerve growth factor induces galectin-3 expression in mouse dorsal root ganglia neurons, thereafter promoting neurite outgrowth and neural cell adhesion [19, 20]. Also, galectin-3 is expressed in astrocytes, microglia, macrophages, dendritic cells, and in activated T and B cells. Growing data are accumulating on the role of galectin-3 in neuroinflammation and neurodegeneration [17]. Gal-3 has been associated with microglial activation in several neuropathological conditions, e.g., traumatic brain injury [21], viral encephalitis [22], ischemia [23], and demyelination [24]. Attenuated inflammatory responses were observed in *galectin-3* deficient mice suffering from EAE, associated with a marked reduction in the number of CNS-infiltrating leukocytes [8]. Also, it was shown that the expression of *galectin-3* is upregulated before motor impairment and that its expression levels persisted in activated microglia throughout disease progression in a murine model of Huntington’s disease [25]. Furthermore, serum galectin-3 is increased in Parkinson’s disease patients and correlates to the Hoehn-Yahr stage [26].

Our data indicate that the dramatic increase in *galectin-3* expression in the spinal cord of EAE mice is associated with the concordant upregulation of *Lgals3* in all the different cell populations taking part in the MS evolution, including the extravasating T helper cells, microglia, oligodendrocytes, and astrocytes. To our knowledge, this is the first report showing the upregulated expression of *galectin-3* in astrocytes in a model of MS. It is known that loss of galectin-3 function inhibits gliogenesis, while galectin-3 overexpression increases it [27]. Also, galectin-3 overexpression increases the percentage of striatal astrocytes generated by the subventricular zone (SVZ), but decreases the percentage of oligodendrocytes [27]. Moreover, galectin-3 immunoreactivity was increased in the perinatal human SVZ and striatum after hypoxia/ischemia [27]. Interestingly, we observed that *Lgals3* expression was not influenced *in vitro* by the exposure of astrocytes to Th1- or Th17-conditioned medium (Supplementary Fig. S2), advocating the involvement of other factors implicated in the crosstalk among the other cell populations within the CNS lesions.

We have to mention that caution should be used when interpreting data from a preclinical model of human disease. Rodent experimental autoimmune encephalomyelitis (EAE) is currently the most common animal model used for investigating MS etiopathogenesis and for the development and testing of novel therapies. Indeed, most of the FDA-approved MS disease-modifying treatments have previously shown efficacy in EAE, including interferon beta, glatiramer acetate, natalizumab, sphingosine 1-phosphate modulators, dimethyl fumarate, and B cell depletion therapies [28]. However, despite being the model that most closely mimics human MS, EAE has some significant flaws. For instance, rodent EAE mostly affects the spinal cord and causes less brain lesions, while MS patients experience lesions in the brain and/or spinal cord. Moreover, the innate and adaptive immune systems of rodents differ from those of humans, which may limit the translatability of some discoveries [28]. Furthermore, the development of EAE requires an external vaccination step, where adjuvants are frequently used to cause sensitization to myelin antigens. These adjuvants contain bacterial components that trigger the innate immune system through the engagement of pattern recognition receptors. In addition, unlike MS, which lacks a specific identifiable antigen, the inducing antigens in EAE are well identified, even though in some, but not most studies, myelin-derived peptides, including peptides of myelin basic protein (MBP), proteolipid protein (PLP), and myelin oligodendrocyte glycoprotein (MOG), have been proposed as a putative target in MS [29]. Therefore, significant differences in the activation and priming of autoreactive T cells may occur between EAE and MS. Also, the CD8+-shift seen in MS lesions contrasts with the predominance of CD4+-T cells found in the majority of EAE models [28].

It is hence clear that there is no single EAE model that is able to mirror the complexity of human diseases and pitfalls occur when preliminary laboratory results are translated into human prematurely.

Despite these limitations, the analysis of a publicly available transcriptomic dataset generated on MS lesions has produced data that are, at least partially, in accordance with those obtained with the EAE model employed in our study. In particular, in comparison to the normal white matter, MS lesions were found to express significantly higher levels of *Lgals3*, and the application of the co-expression modules analysis combined with the gene ontology strongly suggested that the modulation of *Lgals3* occurred in parallel with the involvement of microglia and immune activation in the MS lesions. Further investigations need to be performed in order to ascertain the pattern of expression of *Lgals3* in the different brain cell populations in MS patients and the effect of Lgals3 modulation in each of these cellular populations in disease activity and progression.

It is worth mentioning that, in the present study, we observed a similar pattern of transcriptional modulation between *Lgals3* and *Lgals3bp*. Lgals3bp (also known as 90 K or Mac-2 BP) is a 90-kDa oligomeric glycoprotein that was originally identified as a ligand of the lactose-specific S-type lectin, galectin-3 (formerly Mac-2). *Lgals3bp* expression is induced by viral infection and upon exposure to pro-inflammatory, such as IFN-α, IFN-β, IFN-γ, and TNF-α. Also, a prominent role for Lgals3bp in tumor progression has been revealed, as elevated expression levels of Lgals3bp in serum and tumor tissue positively correlate with poor survival or a more advanced and/or metastatic disease, in most of the human solid cancers (reviewed in [30]). Hence, the combined inhibition of both galectin-3 and Lgals3bp for the treatment of MS could be envisaged.

The accumulating data regarding the involvement of *Lgals3* in MS suggest the potential use of galectin-3 inhibitors in this setting. Up to date, different inhibitors have been produced, such as 33DFTG, which has been shown to be able to reduce corneal neovascularization and fibrosis in preclinical models [31], and TD139, which reduced concanavalin A-induced liver injury [32]. Another galectin-3 inhibitor (a modified citrus pectin) has also been shown to prevent the breakdown of the blood–brain barrier and brain injury in a mouse model of subarachnoid hemorrhage [33]. Given the multiple effects of galectin-3 exerted on the different CNS resident and invading cells, a better understanding of the involvement of galectin-3 during neuroinflammation and neurodegeneration will potentially define galectin-3 as a therapeutic target.

## Conclusions

Overall, with the present study, we are providing a comprehensive view of the expression levels of *galectin-3* in different cellular populations involved in the etiopathogenesis of MS. The significant upregulation of the levels of *Lgals3* in most of the analyzed cell populations and the gene ontology analysis supports the potential pathogenetic role of this galectin in the context of MS. However, whether this modulation of *Lgals3* is a driver of the disease or a consequence of genetic and/or epigenetic factors remains to be determined. Following the accurate identification of the pathways associated with *Lgals3* that underly MS pathogenesis, tailored pharmacological strategies may be propelled toward the treatment of MS patients.

### Supplementary Information

ESM 1

ESM 2

## Data Availability

Data used in this study are available from the corresponding author upon reasonable request.
