# Supplementary material for: Transcriptional upregulation of galectin-3 in multiple sclerosis
Source: Immunol Res. 2023 Jul 25;71(6):950–8. doi: 10.1007/s12026-023-09408-x (PMC10667405; doi:10.1007/s12026-023-09408-x)
Supplement: Supplementary file 1 — ESM 1 [file 12026_2023_9408_MOESM1_ESM.pdf]

Astrocytes (GSE113579)

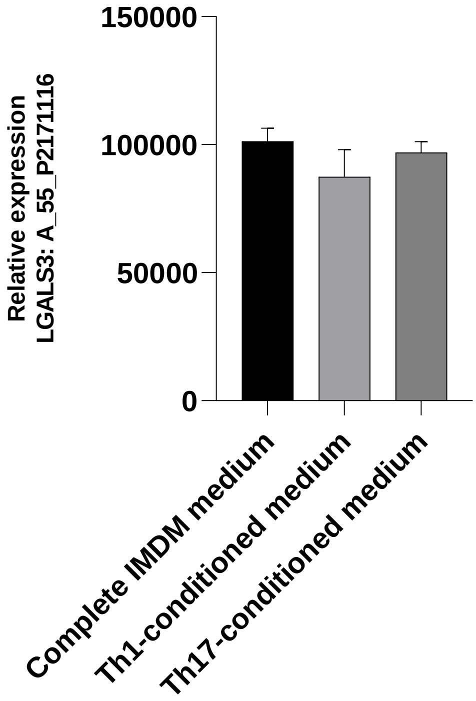

Data were retrieved from the GSE113579 microarray dataset, available on the Gene Expression Omnibus (GEO) database. Primary cultures of mixed glial cells were prepared from brains of neonatal C57BL/6 neonatal mice. Astrocytes were harvested and maintained in culture medium until they reached the confluence. Astrocyte cultures were treated with T cell culture medium (complete IMDM), Th1 or Th17 culture supernatants diluted in equal volume of DMEM 10% FCS for 16h. RNA was isolated from astrocytes and analyzed using the Whole Mouse Genome Oligo Microarray 4x44K v2. Raw data were pre-processed and normalized by quantile normalization followed by global linear scaling. Detailed description of the experimental design can be retrieved from Prajeeth CK et al., 2018 (PMID: 30364000). Statistical differences among experimental groups were assessed using LIMMA and p values adjusted using Benjamini-Hochberg correction. An FDR<0.05 was considered as threshold for statistical significance.
