# Supplementary material for: Transcriptional upregulation of galectin-3 in multiple sclerosis
Source: Immunol Res. 2023 Jul 25;71(6):950–8. doi: 10.1007/s12026-023-09408-x (PMC10667405; doi:10.1007/s12026-023-09408-x)
Supplement: Supplementary file 2 — ESM 2 [file 12026_2023_9408_MOESM2_ESM.pdf]

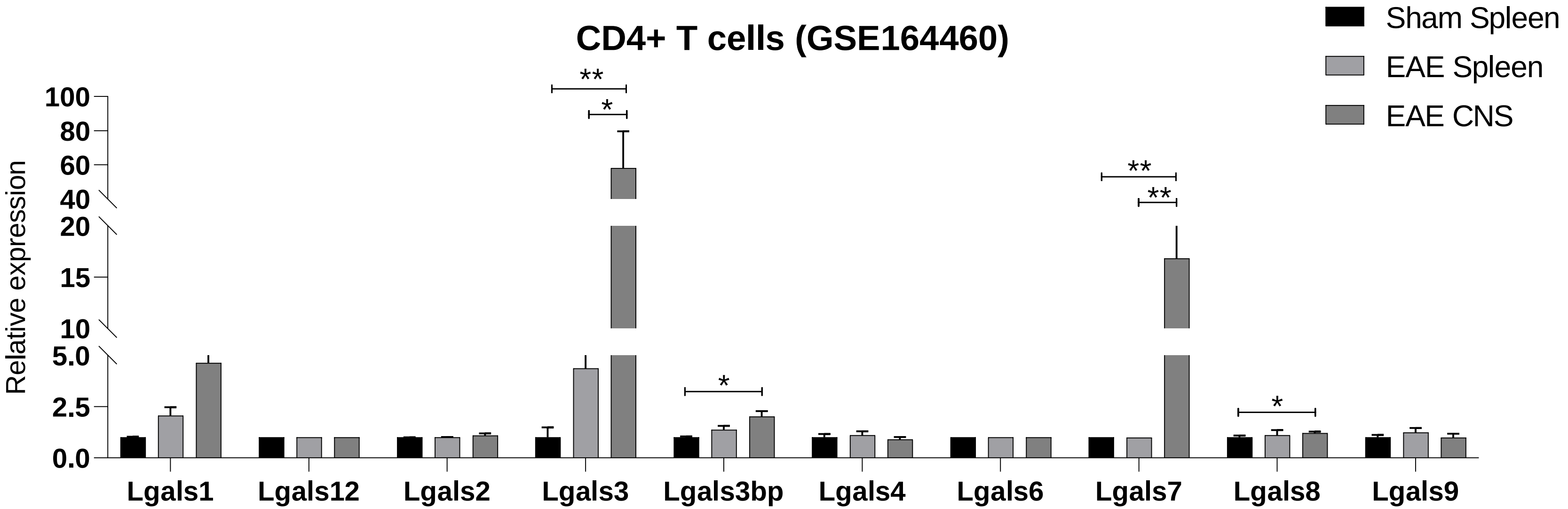

Data were retrieved from the GSE164460 microarray dataset, available on the Gene Expression Omnibus (GEO) database. CD4+ T cells were isolated from the spleen of naïve mice and from the spleen and central nervous system of mice at the peak of experimental autoimmune encephalomyelitis (EAE) induced by immunization with MOG (35-55). RNA was isolated from astrocytes and analyzed using the Affymetrix Mouse Genome 430 2.0 Array. Raw data were pre-processed and normalized using GCRMA. Detailed description of the experimental design can be retrieved from Pohar J et al., 2022 (PMID: 35579560). Statistical differences among experimental groups were assessed using LIMMA and p values adjusted using Benjamini-Hochberg correction. An FDR<0.05 was considered as threshold for statistical significance. *FDR<0.05;**FDR<0.01; ***FDR<0.001.
